# Localized activity attenuates the combined impact of a high fat meal and prolonged sitting on arterial stiffness: A randomized, controlled cross-over trial 

**DOI:** 10.3389/fphys.2023.1107456

**Published:** 2023-02-01

**Authors:** Simon Fryer, Craig Paterson, Louise Turner, Arsalan Moinuddin, James Faulkner, Lee Stoner, Anne Daykin, Keeron Stone

**Affiliations:** ^1^ School of Natural, Social and Sport Sciences, University of Gloucestershire, Cheltenham, United Kingdom; ^2^ Department of Exercise and Sport Science, University of North Carolina, Chapel Hill, NC, United States; ^3^ Department of Sport and Exercise, University of Winchester, Winchester, United Kingdom; ^4^ Department of Epidemiology, Gillings School of Public Health, University of North Carolina, Chapel Hill, NC, United States; ^5^ School of Health and Social Care, University of Gloucestershire, Gloucester, United Kingdom; ^6^ Cardiff School of Sport and Health Sciences, Cardiff Metropolitan University, Cardiff, United Kingdom

**Keywords:** CFPWV, carotid femoral pulse wave velocity, trygliceride-rich lipoproteins, leg fidgeting, uninterrupted sitting, endothelial function

## Abstract

Exposure to acute prolonged sitting and consumption of a high fat (HF) meal have been shown to independently and additively impair central and peripheral cardiovascular function. This study sought to determine whether localized activity, namely leg fidgeting, offers a protective effect to these deleterious effects. Using a randomized crossover design with three trials, 18 healthy males sat uninterrupted for 180 min following the consumption of a low fat (LF, trial 1) or HF meal (trial 2). The third trial consisted of a HF meal but sitting was interrupted with 1 min of leg fidgeting (isolated bilateral plantar flexion) consisting of −250 taps per min every 5 min for the 180 min duration. Carotid-femoral pulse wave velocity (cfPWV), aortic-femoral stiffness gradient (af-SG), superficial femoral blood flow, shear-rate and PWV_β_, triglyceride concentrations and lower-limb venous pooling (HHb) were assessed pre and post sitting in all trials. General linear mixed model found that following the uninterrupted HF trial, there was a significant worsening of cfPWV (mean difference (MD) = 0.57 mˑs^−1^; *d* = 1.04) and the af-SG (MD = 0.14, *d* = 0.50), and femoral artery blood flow (MD = 18 mlˑmin^−1^; *d* = 0.48) and shear rate (MD = 15 S^1^; *d* = 0.67) decreased. However, leg fidgeting was enough to prevent the combined deleterious effects of prolonged sitting following a HF meal. As there were no significant changes in the LF trial, the HF meal maybe the predominant driver when uninterrupted sitting is combined with a HF meal.

## 1 Introduction

There is a growing body of evidence to suggest acute exposure to sedentary behaviours such as prolonged uninterrupted sitting results in transient vascular dysfunction (Credeur et al., 2019; Paterson et al., 2020; Paterson et al., 2021). Uninterrupted sitting for as little as 1 hour induces endothelial dysfunction in the lower limb arteries (Thosar et al., 2014). Whilst uninterrupted sitting for 3 h increases (worsens) aortic pulse wave velocity (PWV) (Credeur et al., 2019) and leads to a reduction in the physiologically advantageous aortic to femoral arterial stiffness gradient (afSG) (Fryer et al., 2021). Given these markers of central and peripheral cardiovascular function are associated with cardiovascular disease risk and mortality (Ben-Shlomo et al., 2014; Stone et al., 2021), understanding their response to sedentary behaviours such as prolonged sitting, and their interaction with other negative lifestyle behaviours is important. One negative lifestyle behaviour of recent interest is the consumption of a high fat (HF) meal prior to prolonged uninterrupted sitting (Cho et al., 2020; Fryer et al., 2021; Coyle et al., 2022). A one off HF meal (50 g) has been shown to increase triglyceride-rich lipoprotein concentrations and reduce postprandial endothelial function by 11% for 2–4 h (Vogel et al., 1997); when combined with 180 min of prolonged uninterrupted sitting, a HF meal (50 g) increased (worsened) carotid-femoral pulse wave velocity (cfPWV) by 0.6 ms^−1^ compared to 0.2 ms^−1^ after a low fat (LF) meal (Fryer et al., 2021). This triglyceride-induced endothelial dysfunction is likely a consequence of acute morphological and cellular changes within the arterial system.

Given that acute prolonged sitting and a HF meal have been shown to independently (Vogel et al., 1997; Credeur et al., 2019) and additively (Fryer et al., 2021) impair central and peripheral cardiovascular function, there is a need to determine whether interrupting sitting with activity, may offer a protective effect to these deleterious effects. Recently, Cho et al. (2020) found that after the consumption of a HF meal, uninterrupted sitting caused a significant decrease in popliteal blood flow and shear rate, and this was improved when stair climbing was used as an interruption strategy. Whilst stair climbing might be a biologically efficacious interruption strategy, the heart rate (HR) data from Cho et al. (2020) suggests it is classified as high-intensity and therefore lacks practicality, and behaviourally may not be possible for everyone to do (Thomas et al., 2015). One potential interruption strategy which may be behaviourally acceptable could be the use of localized isolated plantarflexion activity. Localized isolated plantarflexion activity in this case, is defined as leg fidgeting, and has been shown to be a potent method for: improving systemic metabolic regulation, very low-density lipoproteins triglyceride concentrations (Hamilton et al., 2022), reducing venous pooling in the lower limbs (a potential key driver of sitting induced vascular dysfunction) (Credeur et al., 2019), improving lower limb endothelial function (Morishima et al., 2016), and improving cerebral oxygenation and cognition (Fryer et al., 2022). What is not known is whether leg fidgeting can attenuate the impairment in central and peripheral vascular function following the combination of a HF meal consumption and prolonged uninterrupted sitting. As such, the primary aim of this study was to determine whether interrupting prolonged sitting with frequent leg fidgeting following consumption of a HF meal would mitigate central and peripheral vascular dysfunction.

## 2 Materials and methods

### 2.1 Participants

This study is reported in accordance with the Consolidated Standards of Reporting Trials guidelines (Schulz et al., 2010). Eighteen healthy male participants were recruited. All participants were asymptomatic of any illness, met current United Kingdom physical activity guidelines (Physical Activity Guidlines GOV, 2022), were non-smokers, and were not suffering from any known cardiovascular or metabolic diseases, nor were they taking any known vascular-acting medication. Prior to recruitment and data collection, institutional ethical approval was obtained, which conformed to both the standards of the journal as well as the Declaration of Helsinki, was obtained. All participants gave written informed consent.

### 2.2 Experimental protocol

The experimental protocol comprised of four separate visits to a laboratory, all were conducted within a 10-day period. During visit one, participants had their height, body mass and physical activity status assessed before being familiarized with all experimental procedures and equipment. The three following visits consisted of a HF trial, LF trial and a high-fat fidgeting (HFF) trial, of which the order was randomized (using https://www.randomizer.org/). Each session commenced at 08:30 following an overnight fast, consuming only water and having refrained from strenuous exercise and alcohol for a 24-h period. Prior to the first experimental visit, the evening meal was recorded, and participants were asked to repeat this before subsequent visits.

At the start of each experimental visit, participants were asked to empty their bladder and bowel before quietly lying supine on a test bed for 20 min. During this period, participants were fitted with all equipment including an oscillometric blood pressure cuff (SphygmoCor Xcel, Atcor Medical, Australia) over the upper left arm to determine all pulse wave analysis (PWA) variables. To determine cfPWV and femoral-ankle pulse wave velocity (faPWV), pressure cuffs were placed over the left thigh and ankle respectively. To determine changes in venous pooling over the 180 min sitting periods, a continuous wave near infrared spectroscopy (NIRS- Portalite, Artinis, Netherlands) device was placed over the muscle belly of the dominant gastrocnemius (located using ultrasound).

All ultrasound, PWA and PWV measures were conducted in a supine position. A Doppler ultrasound (T3300, Terrason, Burlington, United States) fitted with a linear array probe (15–4 mHz) was used to collect 3 × 10 s videos of the superficial femoral artery (SFA) on the left side of the body. Immediately after this, and remaining in the supine position, the SphygmoCor Xcel was used to determine PWA variables at the left brachial artery. Following this, determination of cfPWV followed by faPWV occurred; the af-SG was subsequently calculated off-line. After this, the participant was manually moved into a seated position using an electronic three-way tilt table (Plinth 2000; Plinth Medical, United Kingdom). A baseline blood sample for determination of glucose and triglyceride concentration was taken from a fingertip using capillary sampling. From the seated position the participant was given 10 min to eat their breakfast meal. Participants were asked (and continually monitored) to either 1) not aggressively move their lower limbs during the two uninterrupted sitting trials, and 2) perform bilateral heel raises for 60 s of every 5-min period of the 180 min during the interrupted sitting trial. During both uninterrupted trials the feet were supported by a foam block to ensure the participant was comfortable throughout. If a participant needed to urinate, specially designed trousers with multiple zips allowed for ease of access so urination could take place in private without any aggressive lower limb movement. No participants needed to empty their bowels during any testing sessions. Following 180 min of sitting, triglyceride samples were repeated before the participants were lowered back to a supine position for a 10 min period of quiet rest. Following this, all cardiovascular assessments were repeated in the same order as the baseline measures.

### 2.3 Experimental procedures

#### 2.3.1 Meal types

In accordance with previous research looking at understanding the effects of a HF meal on vascular function (Vogel et al., 1997), we used a McDonald’s Corporation breakfast meal which included a double sausage and egg McMuffin, two hash browns and a hot chocolate with added double cream (1,066 kcal, 4.5 MJ, 61 g fat [of which 20 g was saturated fat], carbohydrates 86 g, protein 40 g and salt 5 g). A one-off meal using a difference of 50 g of fat (LF vs. HF trails) has previously been shown to independently cause endothelial dysfunction in healthy individuals (Vogel et al., 1997). The LF meal consisted of two large English crumpets (Kingsmill Inc. United Kingdom) each with 10 g of low-fat spread (Tesco PLC. United Kingdom), 5 g of Marmite (Marmite, Unilever, United Kingdom), and 200 mL skimmed milk beverage with 22 g of unflavoured whey protein powder (Impact Whey, MyProtein, United Kingdom) (601 kcal, 2.5 MJ, 10 g fat [of which 3 g was saturated fat], 86 g carbohydrate, 40 g protein, 5 g salt).

#### 2.3.2 Localized activity

Leg fidgeting was the localized activity used to interrupt the 180 min sitting period. Participants were asked to perform bilateral heel raises (isolated plantar flexion) at a beat of a metronome at −250 taps per min, for 1 min on/4 min off, for the entire 180 min sitting period. Previously this technique and timing, has been shown to improve lower limb blood flow and shear rate (Morishima et al., 2016). A member of the research team informed the participant when to start and stop each 1 min bout of fidgeting.

#### 2.3.3 Pulse wave velocity

The SpygmoCor XCEL device enables simultaneous assessment of proximal and distal arterial waveforms to determine arterial pulse transit time (PTT) using a tonometer and volume displacement cuff respectively. PWV is calculated by dividing arterial path length by PTT, or PWV distance/time. For cfPWV, the tonometer was placed on the left carotid artery and the oscillometric cuff was placed on the left thigh at the level of the femoral artery, following recommended manufacturer guidelines (Butlin et al., 2013). Using custom made callipers, the carotid-femoral *D* was estimated by measuring the linear distance from the suprasternal notch to the top of the cuff at the centre line of the leg and subtracting the distance from the suprasternal notch to the carotid artery (the subtraction method) in accordance with procedures recommended by the American Heart Association (Townsend et al., 2015). For faPWV, the tonometer was placed at the point of maximal pulsation (obtained by palpation) at the level of the superficial femoral artery (SFA), whilst the ankle cuff (SC10, Hokanson) was positioned with the bottom edge proximal to the malleolus. Femoral-ankle *D* was estimated by measuring the linear distance from the point of tonometric applanation to the top of the ankle cuff at the centre line of the leg. Femoral-ankle PTT was corrected prior to the calculation of PWV as previously described (Stone et al., 2019). All PWV measures were assessed in triplicate as a minimum, and quadruplicate as a maximum, with at least two measures being between 0.3 mˑs^−1^ of each other. The average of the closest two measures were used in all analysis. The American Heart Association recommendations for improving and standardizing vascular research on arterial stiffness (Townsend et al., 2015) suggests PWV within 0.5 mˑs^−1^ is considered excellent for comparisons. However, our study chose 0.3 mˑs^−1^ as a more conservative figure given the expected changes in PWV caused by sitting with a HF meal were less than 1 mˑs^−1^.

#### 2.3.4 Local pulse wave velocity, blood flow, and shear rate

Local measures of SFA PWV_*ß*_, blood flow, and shear rate provide additional mechanistic information which complement regional arterial stiffness measures when determining the effects of prolonged sitting on arterial function (Fryer et al., 2019). A trained ultrasound operator with extensive experience ensured that the vessel was extended across the entire (un-zoomed) imaging plane to minimize the risk of skewing the vessel walls. Ultrasound global (acoustic output, gain, dynamic range, gamma and rejection) and probe-dependent (zoom factor, edge enhancement, frame averaging and target frame rate) settings were standardized. Three 10 s videos of the ultrasound readings were recorded using external video capturing software (LiteCam, LiteCam HD, United States). During each 10 s video capture, participants were instructed to hold their breath (without having a large inhalation).

The video clips were analyzed offline using automated edge-detecting software (Quipu, FMD Studio, Italy) by a trained operator blinded to the condition. Custom written Excel Visual Basic code was used to fit peaks and troughs to the diameter waveforms in order to calculate diastolic, systolic, and mean diameters. Blood flow was calculated from continuous diameter and mean blood velocity recordings using the equation: 3.14 x (diameter/2)^2^ x mean blood velocity x 60. A local, single-point measure of PWV was calculated using the PWV_*ß*_ equation, described below.

(1) The *ß*-stiffness derivative method utilizes the *ß-*stiffness index to estimate PWV. The *ß-*stiffness index is based on changes in pressure and diameter and can be described as: PWV_*ß*_ *=* $\sqrt{\left(ß\cdot DBP\right)/\left(2p\right)}$

Where; *p* is the blood density (1,059 kg/m^3^) (Harada et al., 2002) and *ß* is the *ß*-stiffness index, which is calculated using the formula:

$$\beta =\ln\left(SBP/DBP\right)/\left[\left(Ds-Dd\right)/Dd\right]$$

where ln is the natural logarithm, SBP is systolic blood pressure, DBP is diastolic blood pressure, Ds is the lumen diameter during systole, and Dd is the lumen diameter during diastole (Kawasaki et al., 1987). In accordance with Thijssen, Bruno (Thijssen et al., 2019) mean blood velocity was estimated by taking half of peak velocity. As the arterial sample volume was large, shear rate was calculated as 8 x mean blood velocity/internal diameter (Thijssen et al., 2019). For transparency, unknown errors in two ultrasound videos from the HF trial were not able to be read and so all their respect ultrasound data was removed. Local SFA measures are reported as n = 16.

### 2.4 Pulse wave analysis

The SphygmoCor Xcel was used to measure brachial blood pressure and conduct PWA to determine central pressure hemodynamics pre and post 180 min of sitting. An appropriately sized cuff was selected according to manufacturer guidelines and placed around the upper left arm. The upper arm cuff was initially inflated for approximately 30 s to measure brachial systolic blood pressure (SBP) and diastolic blood pressure (DBP), and then immediately re-inflated to 10 mmHg below DBP to acquire a volumetric displacement signal for 10 s. The brachial waveforms were calibrated using the cuff measured SBP and DBP, and mean arterial pressure (MAP) was derived by integrating the area under the curve. A corresponding aortic pressure waveform was generated using a validated transfer function and calibrated using DBP and MAP (Butlin et al., 2012). The aortic waveform was used to derive central: systolic blood pressure (cSBP), diastolic blood pressure (cDBP), mean arterial pressure (MAP), pulse pressure (cPP), augmentation pressure (cAP), augmentation index (AIx), augmentation index normalized to a heart rate of 75 bpm (AIx@75), forward aortic pressure (Pf), backward aortic pressure (Pb) and the Buckberg subendocardial viability ratio (SEVR) were derived. The Artery Task Force suggest central blood pressure may influenced by respiration by 2–4 mmHg (Sharman et al., 2017). As such, all PWA assessments were conducted in triplicate as a minimum, and quadruplicate if variability in cSBP was >4 mmHg. For all PWA variables, the average of the closest two were used for all analyses. In accordance with Sharman (Sharman et al., 2017) each PWA assessment was separated by a 1-min period.

### 2.5 Blood sampling

Glucose (mmol·L) was sampled using a Biosen C_Line (Biosen-C line, EKF Diagnostics, Wales) at the start of each testing session as an indicator of adherence to the overnight fasting prerequisite, and as a screening for potential diabetes. Triglyceride (mg·dL) samples were analyzed using a Reflotron (Reflotron, Hoffmann-La Roche LTD., Switzerland) pre and post the 180 min sitting periods; peak postprandial triglyceride concentrations have been shown to occur between 2 and 12 h after a high fat meal (Cohn, 2006). The Reflotron and Biosen had an intra-precision of 5.2% and 1.27% respectively (Von Schenck et al., 1987; Nowotny et al., 2012). For both glucose and triglycerides, a 1.6 mm blood lancet (Safe T Plus, Accu-Chek, Switzerland) punctured the fingertip, all blood samples were collected from the capillary using a 20 μL (glucose) and 32 μL (triglyceride) lithium heparin capiliette (Sarstedt, Aktiengesellschaft & Co., Germany).

### 2.6 Near infrared spectroscopy

Continuous-wave NIRS was used to determine changes in venous blood volume (deoxy-hemoglobin (HHb)) in the gastrocnemius as a measure of blood pooling, pre- and post-180 min of uninterrupted and interrupted sitting. NIRS relies on the relative transparency of muscle tissue to infrared light, and the absorption characteristics of hemoglobin (Hb) to determine oxy-hemoglobin (O_2_Hb) and HHb, the sum of which is total hemoglobin (tHb). Changes in HHb using NIRS have previously been shown to be both valid and reliable during an orthostatic challenge (Stone et al., 2016). It should be noted that as NIRS cannot distinguish between O_2_Hb and oxy-myoglobin; for clarity this paper will refer to the combination of both as Hb.

### 2.7 Sample size

Using the effect size of η_p_^2^ = 0.64 derived from the main effect of time for change in cfPWV between pre-and post-180 min of sitting (Fryer et al., 2021) and the maximum chances of type 1 error set at 5% (i.e. very unlikely) and power set at 0.95, the approximate number of participants required using G*Power, was 15. However, 18 were recruited to account for the unknown combined effects of localized exercise, a HF meal and prolonged sitting on hemodynamic parameters.

### 2.8 Statistical analysis

Statistical analyses were conducted using Jamovi (Version 1.8), a graphical front end to the R programming language, using the general analysis for linear mixed models (GAMLj) module (Jamovi), and one way ANOVA. Raw data are presented as mean ± SD, and mixed model data is presented as mean difference with 95% confidence intervals. Follow-up analyses were conducted using Bonferroni correction. The alpha level of significance was set at ≤ 0.05 for all analyses. Given the interdependence of BP on arterial stiffness, cfPWV and faPWV were adjusted for MAP. Cohens’*d* is reported as a measure of effect sizes where ˂0.2, 0.2, 0.5, and ≥0.8 were considered trivial, small, moderate, and large respectively (Cohen, 2013).

## 3 Results

### 3.1 Participant demographics

The mean (SD) for age, height, weight and BMI of all 18 participants were 21.7 (1.9) yrs; 178.4 (5.5) cm; 76.9 (8.9) kg; 24.2 (2.8) kg∙m^2^ respectively.

### 3.2 Pulse wave velocity

The interaction and main effects for cfPWV, faPWV, and the af-SG are presented in Table 1. Following a significant time × condition interaction for cfPWV, *post hoc* analyses revealed it significantly increased in the HF trial (MD = 0.57 ms^−1^, 95% CI 0.33–0.80, *p* < 0.001, *d* = 1.04), but not in the LF (MD = 0.21 ms^−1^, 95% CI 0.02–0.44, *p* < 0.081, *d* = 0.28), or HFF trials (MD = 0.11 ms^−1^, 95% CI 0.14–0.35, *p* = 0.397, *d* = 0.09). There were no significant interaction, group or time effects for faPWV. There was a significant main effect time for the af-SG, following Bonferroni correction the af-SG significantly decreased over time in the HF trial only (MD = 0.14, 95% CI 0.04–0.23, *p* = 0.007, *d* = 0.50).

**TABLE 1:** Mean (±SD) interaction and main effects for central and peripheral pulse wave velocity, and the aortic-femoral stiffness gradient pre and post low fat, high fat and high fat fidgeting sitting trials (*n* = 18).

		cfPWV (m·s^−1^)	faPWV (m·s^−1^)	Af-SG
Mean average (SD)				
**Low Fat**	0 min	5.65 (0.70)	9.65 (1.0)	1.65 (0.20)
	180 min	5.83 (0.58)	9.61 (1.14)	1.60 (0.24)
**High Fat**	0 min	5.55 (0.48)	9.27 (1.33)	1.65 (0.25)
	180 min	6.09 (0.56)	9.13 (1.17)	1.52 (0.27)
**High Fat Fidget**	0 min	5.59 (0.61)	9.09 (1.21)	1.60 (0.26)
	180 min	5.64 (0.45)	9.09 (1.02)	1.57 (0.27)
**Interaction Effect**				
	*p*	0.010	0.657	0.330
**Time Effect**				
	*p*	<0.001	0.662	0.010
**Condition Effect**				
	*p*	0.220	0.098	0.430

SD, standard deviation; *p*, significance; cfPWV, carotid to femoral pulse wave velocity; faPWV, femoral to ankle pulse wave velocity; m·s^−1^, meters per second; min, minute; af-SG, aortic-femoral stiffness gradient.

### 3.3 Local femoral artery measures

As presented in Table 2, the only significant interaction effect for measures assessed in the SFA were for blood flow and shear rate. Following Bonferroni correction, there was a significant reduction in blood flow (MD = 18 mL min^−1^, 95% CI 8–82, *p* = 0.019, *d* = 0.48) and shear rate (MD = 15 S^1^, 95% CI 3–26, *p* = 0.011, *d* = 0.67) following the HF trial only. There was a significant main effect of time for PWV_β_ only however, following Bonferroni correction no individual trial significantly changed over time.

**TABLE 2:** Mean (±SD) interaction and main effects for ultrasound derived superficial femoral artery measures pre and post low fat, high fat and high fat fidgeting sitting trials (*n* = 16).

	Superficial femoral artery					
		Blood flow (ml·min)	Shear rate (S^−1^)	Avg. Diameter (mm)	Velocity (cm·s^−1^)	PWV_ß_ (m·s^−1^)
Mean average (SD)						
**Low Fat**	0 min	314 (138)	39.9 (22.90)	6.73 (1.01)	15.0 (3.70)	5.72 (1.11)
	180 min	287 (126)	31.8 (16.50)	6.67 (1.05)	15.2 (2.90)	6.32 (1.44)
**High Fat**	0 min	329 (106)	50.6 (29.70)	6.68 (0.98)	16.1 (4.40)	5.99 (1.65)
	180 min	284 (98)	36.0 (31.20)	6.62 (1.03)	16.0 (4.50)	6.11 (1.06)
**High Fat Fidget**	0 min	340 (113)	43.0 (23.50)	6.76 (0.97)	16.8 (4.10)	5.76 (0.28)
	180 min	360 (113)	49.7 (19.90)	6.77 (1.07)	17.1 (3.50)	6.29 (1.02)
**Interaction Effect**						
	*p*	0.043	0.032	0.814	0.825	0.516
**Time Effect**						
	*p*	0.118	0.109	0.465	0.623	0.026
**Condition Effect**						
	*p*	<0.001	0.071	0.173	0.018	0.992

SD, standard deviation; m·s^−1^, meters per second; cm·s^−1^, centimetres per second; PWV_ß_, pulse wave velocity beta; S^−1^, Shear; mm, millimetres; ml·min, millilitres per minute; min, minute.

### 3.4 Venous pooling (tHb), and blood concentration

Table 3 presents a significant interaction effect for both triglyceride concentration, and venous pooling (HHb) in the gastrocnemius. Following Bonferroni correction triglyceride concentration significantly increased in both the HF (MD = 67 mg dL, 95% CI 49–85, *p* < 0.001, *d* = 1.7) and HFF (MD = 46.7 mg dL, 95% CI 28–65, *p* < 0.001, *d* = 1.3) trials. Following Bonferroni correction HHb concentration, a marker of venous significantly increased in the HF (MD = 6 μmol, 95% CI 3–9, *p* < 0.001, *d* = 0.99) and LF (MD = 5 μmol, 95% CI 2–9, *p* = 0.002, *d* = 0.89) trials but not the HFF. Whilst there was no significant interaction effect for calf circumference, there was a significant time effect with Bonferroni correction revealing a significantly increased circumference over time in the HF (MD = 1.5 cm, 95% CI 1.1–2.0, *p* < 0.001, *d* = 0.52), LF (MD = 1.5 cm, 95% CI 1.1–1.9, *p* < 0.001, *d* = 0.56) and HFF (MD = 0.9 CM, 95% CI 0.5–1.3, *p* < 0.001, *d* = 0.28) trials. A one-way ANOVA found no differences in fasting glucose concentrations between the LF (mean = 4.40, SD = 0.52 mmolˑL), HF (mean = 4.27, SD = 0.8 mmolˑL), or HFF (mean = 4.77, SD = 0.54 mmolˑL) trials; no fasted sample was >7 mmolˑL and as such no one was considered pre-diabetic (Goldenberg and Punthakee, 2013).

**TABLE 3:** Mean (±SD) interaction and main effects hemoglobin markers pre and post low fat, high fat and high fat fidgeting sitting trials (n = 18).

		Triglyceride (mg·dL)	Calf circumference (cm)	Venous pooling (µmol)
Mean average (SD)				
**Low Fat**	0 min	71.0 (4.20)	37.7 (2.70)	13.6 (6.21)
	180 min	86.6 (23.50)	39.2 (2.70)	19.0 (5.92)
**High Fat**	0 min	75.0 (9.10)	37.8 (2.90)	11.9 (5.63)
	180 min	142.0 (53.60)	39.3 (2.90)	17.7 (6.11)
**High Fat Fidget**	0 min	73.3 (8.60)	37.9 (2.90)	13.2 (6.29)
	180 min	120.0 (52.30)	38.7 (2.90)	12.0 (5.18)
**Interaction Effect**				
	*p*	0.001	0.062	0.003
**Time Effect**				
	*p*	<0.001	<0.001	<0.001
**Condition Effect**				
	*p*	<0.001	0.611	0.024

*p*, significance; SD, standard deviation; min, minute; mg·dL, milligrams per decilitre; cm, centimetres; HHb, deoxygenated hemoglobin.

### 3.5 Pulse wave analysis

There were no significant interactions, or group effects for any PWA variables presented in Table 4. However, there was time effect for HR, AIx, Pf, and SEVR. Post hoc analysis revealed that following Bonferroni correction, HR significantly increased in both the HF (MD = 2 bts·min^−1^, 95% CI 0.4–5, *p* = 0.049, *d* = 0.25) and HFF (MD = 5 bts·min^−1^, 95% CI 3–8, *p* < 0.001, *d* = 0.65) trials but not the LF. Following Bonferroni correction, AIx significant decreased over time in HF (MD = 6, 95% CI 2–9, *p* < 0.001, *d* = 0.75) and HFF (MD = 4, 95% CI 1–8, *p* = 0.016, *d* = 0.44) trials but not the LF. For both the variables Pf and SEVR, following Bonferroni correction there were no significant time differences in any condition.

**TABLE 4:** Mean (±SD) interaction and main effects for all pulse wave analysis measures assessed pre and post low fat, high fat and high fat fidgeting sitting trials (*n* = 18).

		Heart rate (bts·min)	SBP (mmHg)	MAP (mmHg)	cSBP (mmHg)	DBP (mmHg)	cPulse pressure (mmHg)	AIx (%)	AIx@75 (%)	Pf (mmHg)	Pb (mmHg)	SEVR (%)
Mean average (SD)												
**Low Fat**	0 min	56 (6)	114 (9)	78 (6)	100 (6)	65 (6)	34 (6)	4 (8)	−5 (8)	25 (4)	13 (8)	168 (21)
	180 min	58 (6)	115 (7)	76 (7)	100 (5)	65 (4)	35 (7)	3 (7)	−5 (7)	27 (4)	12 (2)	163 (19)
**High Fat**	0 min	58 (9)	117 (7)	78 (5)	101 (6)	64 (5)	38 (7)	6 (8)	−2 (8)	27 (4)	13 (2)	161 (25)
	180 min	60 (10)	117 (10)	78 (5)	100 (7)	64 (6)	36 (11)	1 (7)	−7 (8)	29 (6)	12 (3)	158 (28)
**High Fat Fidget**	0 min	56 (7)	113 (9)	76 (5)	99 (6)	63 (6)	35 (7)	7 (10)	−2 (11)	27 (6)	12 (3)	161 (25)
	180 min	61 (9)	114 (8)	76 (6)	98 (6)	64 (8)	34 (6)	3 (9)	−4 (11)	28 (5)	12 (2)	152 (23)
**Interaction Effect**												
	*p*	0.212	0.884	0.524	0.830	0.491	0.417	0.175	0.290	0.762	0.856	0.710
**Time Effect**												
	*p*	<0.001	0.331	0.794	0.535	0.615	0.759	<0.001	0.052	0.042	0.266	0.045
**Condition Effect**												
	*p*	0.380	0.078	0.197	0.066	0.230	0.083	0.451	0.287	0.053	0.674	0.053

SD, standard deviation; *p*, significance; mmHg, pressure; bts·min^−1^, beats per minute; mmHg = millimetre of mercury; AIx , augmentation index, Pf = pressure forwards, Pb, pressure backwards; bts·min, beats per minute; SEVR, subendocardial variability ratio; SBP, systolic blood pressure; MAP, mean arterial pressure; cSBP, central systolic blood pressure; cPP, central pulse pressure.

## 4 Discussion

The aim of this study was to determine whether interrupting prolonged sitting with frequent leg fidgeting following consumption of a HF meal would mitigate central and peripheral vascular dysfunction. The main findings were that 1) cfPWV and the af-SG were both significantly impaired during the uninterrupted HF sitting trial, and our data suggests that this can be mitigated by using frequent leg fidgeting as an interruption strategy. 2) The changes in cfPWV and af-SG were matched by a significant reduction in both SFA blood flow and shear rate. 3) Independent of condition, 180-min of sitting induced an increase in SFA PWV_β_, and circumference of the gastrocnemius. 4) The LF and HF trials caused an increase in venous pooling (HHb), and this was mitigated in the presence of leg fidgeting.

### 4.1 Strengths and limitations

First, the current study used habitually active male participants and as such the findings cannot be generalized beyond this population. However, our laboratory is currently working hard to address this issue, focusing on several female only studies. Second, participants were asked to ensure that their meal the night before each trial was consistently similar between trials. However, we cannot guarantee that this occurred, but participants were reminded after each trial and food diaries were used to aid compliance. Third, we did not measure participants adiposity and as such we cannot determine the effect this may have had on our primary and secondary outcome measures. Lastly, our study was not sufficiently powered to adjust for multiple potentially confounding variables such as HR, triglycerides and glucose. As such future research should look to conduct similar studies with a larger sample. A significant strength of the study was that all PWV, PWA and ultrasound measures were taken in accordance with the relative published PWV guidelines (Stoner et al., 2011; Stoner and Young, 2012; Stone et al., 2019; Thijssen et al., 2019; Stoner et al., 2021), which can be difficult in sitting research. Further, the study design and data handling were conducted in accordance with Cochrane’s risk of Bias (Sterne et al., 2019), i.e. appropriate counter balanced randomization techniques were used, the data extraction and analysis was conducted by a laboratory member blinded to the trial and time point, and the order of the first two trials were hidden from participants until the start of the trial. Lastly a notable strength of the study design was that this was the first known study to use a LF control trial to compare the effects of consuming a HF meal prior to interrupting sitting.

### 4.2 Comparison to literature

Our data shows that cfPWV and the novel marker af-SG are both impaired to a greater extent following a HF meal in conjunction with prolonged uninterrupted sitting (Table 1), compared to uninterrupted with a LF meal. Of importance, frequent leg fidgeting attenuates this additional burden caused by the HF meal. Previously, Kelsch (Kelsch et al., 2021) assessed the effects of LF beverages with different glycemic indexes on cfPWV following 180 min of prolonged uninterrupted sitting. Similarly, the authors reported no significant changes in cfPWV; changes in arterial stiffness were only found when PWV from several arterial sections were combined to create a global pulse wave velocity score (pre vs. post change = 0.29 mˑs^−1^). As such, it maybe that at least acutely, the sitting induced changes in arterial stiffness are compounded by, and largely driven by the consumption of the HF meal. In support of this, only during the HF trial were SFA blood flow and shear rate significantly reduced (Table 2). The reduction in shear rate and consequently blood flow might be explained by two intertwined theories. Firstly, the lack of muscle pump in the uninterrupted trials would reduce shear rate and blood flow leading to the increase in venous pooling in the gastrocnemius (seen in Table 3). Secondly, whist this increased pooling in the LF trial may not have been enough to impair cfPWV on it’s own, in the presence of higher circulating triglyceride concentrations during the HF trial, their combination may have caused the significant impairment in cfPWV and af-SG *via* acute triglyceride induced morphological and cellular changes. Previously, Vogel, Corretti (Vogel et al., 1997) found that independent of sitting, a HF meal (50 g) increased serum triglycerides and this impaired endothelial function (FMD) by 11%.

Another interesting finding of the present study is that whilst the HF meal appears to be driving a notable portion of the sitting induced dysfunction, when leg fidgeting is used to as an interruption strategy, this impairment is attenuated. Leg fidgeting prevented venous pooling in the gastrocnemius, and SFA shear rate and blood flow were also maintained using this localized activity. The present study used the same leg fidgeting protocol as Morishima, Restaino (Morishima et al., 2016), who found it mitigated the reduction in endothelial function following uninterrupted sitting when meal consumption was not controlled for. However, the current study is the first to show that consumption of a HF meal prior to uninterrupted prolonged sitting compounds sitting induced vascular dysfunction, and that this can be significantly offset when 1 min of rapid leg fidgeting is performed for every 5 min of the sitting period. The ability of leg fidgeting to off-set this vascular dysfunction may be due to the unique oxidative properties of the soleus muscle, which is −88% type I slow-twitch muscle fibre (Johnson et al., 1973). In rat models, the soleus has a phenotype which favours greater uptake of plasma triglycerides compared to other leg muscles (Bey and Hamilton, 2003). Further, the soleus has distinctive vascular features which enhance the delivery of blood-borne fuels and oxygen (McDonough et al., 2005). Hamilton, Hamilton (Hamilton et al., 2022) found that using leg fidgeting in a seated position for a 3-h period, very low-density lipoprotein (VLDL) plasma concentrations were significantly reduced. While VLDLs were not assessed in this study, triglyceride concentration was 32% lower in the HFF compared to the HF trial, and so it may in part explain an attenuated cellular response that underpins endothelial dysfunction. As such, it appears that leg fidgeting may be a powerful tool in offsetting the dysfunction caused by consuming HF meals prior to uninterrupted sitting.

### 4.3 Implications

It appears that a HF meal may be a greater stimulus for vascular dysfunction than prolonged uninterrupted sitting alone. Addressing this should be a key target when clinicians, researchers and policymakers look to intervene with lifestyle behaviours to improve cardiovascular health. Even local activity of the gastrocnemius and soleus is enough to offset the combined effects of a HF meal in conjunction with 180-min of prolonged sitting. For much of the population this localized lower limb activity is simple, easy to do and requires no special equipment. This may be particularly relevant for those with limited mobility or those who cannot regularly interrupt prolonged sitting with whole-body physical activity such as stair climbing or walking. It may also be an efficacious option for desk-based workers.

The lack of significant changes over time within specific conditions of the current study may be caused by a lack of statistical power, as our primary outcome was cf-PWV and not SEVR, Pf or PWV_β_. It is important that future research with larger cohorts should investigate the combined effects of prolonged sitting with HF meals on these other hemodynamic parameters. A further implication for future research which aims to better understand sitting induced vascular dysfunction, is to consider controlling participants nutrition prior to sitting studies as meals high in fat appear to inflate the level of dysfunction posed by uninterrupted sitting alone.

## Data Availability

The raw data supporting the conclusion of this article will be made available by the authors, without undue reservation.
